# An Assessment of the Currently Available Molecular Assay for the Diagnosis of Anisakis Sensitization

**DOI:** 10.3390/ijms26073033

**Published:** 2025-03-26

**Authors:** Maria Barrale, Walter Mazzucco, Santo Fruscione, Maurizio Zarcone, Vincenzo Cantisano, Gaetano Cammilleri, Antonella Costa, Vincenzo Ferrantelli, Rosa Onida, Enrico Scala, Danilo Villalta, Carina Gabriela Uasuf, Ignazio Brusca

**Affiliations:** 1 U.O.C di Patologia Clinica Ospedale Buccheri La Ferla FBF, 90123 Palermo, Italy; vincenzo.cantisano@libero.it (V.C.); onida.rosa@fbfpa.it (R.O.); brusca.ignazio@fbfpa.it (I.B.); 2 U.O.C. di Epidemiologia Clinica con Registro Tumori Azienda Ospedaliera Universitaria Policlinico “Paolo Giaccone”, 90127 Palermo, Italy; walter.mazzucco@unipa.it (W.M.); santo.fruscione@unipa.it (S.F.); maurizio.zarcone@policlinico.pa.it (M.Z.); 3 Istituto Zooprofilattico Sperimentale della Sicilia, 90129 Palermo, Italy; gaetano.cammilleri@izssicilia.it (G.C.); antonella.costa@izssicilia.it (A.C.); vincenzo.ferrantelli@izssicilia.it (V.F.); 4 UOS di Allergologia Molecolare Clinica e di Laboratorio Istituto Dermatologico dell’Immacolata, IDI-IRCCS, 00167 Rome, Italy; e.scala@idi.it; 5 SC Immunologia e Allergologia di Laboratorio Ospedale Santa Maria degli Angeli, 33170 Pordenone, Italy; danilo.villalta@asfo.sanita.fvg.it; 6 Istituto di Farmacologia Traslazionale (IFT), Consiglio Nazionale delle Ricerche (CNR), 90146 Palermo, Italy; carinagabriela.uasuf@ift.cnr.it

**Keywords:** Anisakis, Ani s 3 allergen, tropomyosin, macroarray analysis, food hypersensitivity

## Abstract

The diagnosis of allergic reactions to *Anisakis* remains challenging due to the lack of specific allergens available for routine clinical use. However, the latest version of the multiplex macroarray ALEX-2 now allows the detection of specific IgE against Ani s 1, the major species-specific allergen, as well as Ani s 3 (tropomyosin), a highly cross-reactive molecule with homologs in other allergenic sources. This study aimed to evaluate the potential role of ALEX-2 in diagnosing *Anisakis* sensitization by comparing it with a previously validated diagnostic algorithm. Serum samples from patients with suspected *Anisakis* sensitization were consecutively collected at an Italian allergy centre. Diagnosis was based on a history of allergic reactions following seafood consumption, along with negative test results for fish allergy. All patients underwent skin prick testing and specific IgE measurement for *Anisakis* (p4), *Ascaris* (p1), shrimp (f24), and *Dermatophagoides pteronyssinus* (d1), as well as tropomyosins from house dust mites (d205) and shrimp (f351). Additionally, the basophil activation test (BAT) using crude *Anisakis* extract was performed. Patients were also tested using the ALEX-2 allergy macroarray. Correlation analyses and multiple logistic regression models were applied to assess associations between conventional singleplex tests and the severity of clinical manifestations. A total of 33 eligible subjects were recruited, including 20 females (60.6%). Seven (21.2%) were aged 0–29 years, eleven (33.3%) were 30–60 years old, and fifteen (45.5%) were over 60 years old. ALEX-2 showed positivity for Ani s 1 or Ani s 3 in 39.39% (95% CI: 22.90–57.86%) of subjects with confirmed *Anisakis* sensitization. A significant correlation was observed between Ani s 3 (r = 0.31 [95% CI: 0.04–0.56], *p* = 0.01) and *Ascaris* (r = 0.35 [95% CI: 0.129–0.55], *p* = 0.004) levels and the severity of clinical reactions. Despite the limitations of this cross-sectional study, including a small sample size, our preliminary findings suggest that the ALEX-2 macroarray may not be sufficiently sensitive for the first-line diagnosis of *Anisakis* allergy. However, it could provide valuable additional information, as Ani s 1 positivity indicates primary sensitization to the nematode, while Ani s 3 positivity appears to correlate with clinical severity. Larger prospective longitudinal studies are needed to confirm these findings and further assess the predictive value of ALEX-2 in diagnosing *Anisakis* allergy.

## 1. Introduction

*Anisakis* spp. belong to the subfamily *Anisakinae*, family *Anisakidae*, superfamily *Ascaridoidea,* and order *Ascarida* [1,2]. Humans are not definitive hosts but can also be accidental hosts for Anisakis, which is unable to complete its life cycle in humans. The nematode can cause various clinical manifestations due directly to parasitic infection (Anisakiasis) or to hypersensitivity allergic reactions [3]. Patients can simultaneously exhibit symptoms of infestation and those of allergic sensitization, as occurs in the gastro-allergic clinical form. Contact with the parasite can lead to allergic sensitization towards different antigenic epitopes [3]. The clinical symptoms are determined by the location of the parasite in the gastrointestinal tract and the extent of the allergic manifestations [4]. Once sensitization has occurred, even after the infestation has been resolved, allergic reactions can occur upon ingestion of seafood products, even if cooked, containing the parasite [5], with symptoms ranging from hives to anaphylactic shock. The prevalence of *Anisakis*-induced allergies is highly dependent on dietary habits, with a higher incidence in populations where raw fish is commonly consumed [4,6]. Workers in the fish industry are also at increased risk of sensitization [3,7]. The diagnosis of *Anisakis* infestation is endoscopic, with the direct identification of larvae in the digestive tract. Although endoscopy is the best option for diagnosing anisakiasis, the determination of specific IgE is currently the most common clinical practice, as it is sometimes difficult to identify these cases through anamnesis. The diagnosis of *Anisakis* allergy is much more challenging. Indeed, it requires distinguishing between a specific allergy to ingested seafood, a toxic reaction such as scombroid syndrome, and an allergy to the nematode itself.

Diagnosing *Anisakis* allergy is challenging due to the presence of numerous pan allergens in *Anisakis* extracts, making it difficult to distinguish genuine sensitization from cross-reactivity with other allergens. As a result, positive IgE or skin prick tests for *Anisakis* can give false positive results due to the cross-reactivity with other sensitizing agents such as house dust mites, crustaceans, and other nematodes [4,8,9]. Additionally, a high prevalence of IgE against the parasite has been observed in patients with chronic spontaneous urticaria, with approximately 24% showing positivity. However, only one-third of these cases have a direct causal link, where *Anisakis* acts as a real trigger for skin manifestations [10,11]. Patients with chronic urticaria may show serological positivity for nematodes that only in some cases seem to be related to *Anisakis* ingestion. In fact, many of them do not respond to a prolonged fish-free diet in areas with high infestation rates [12]. Furthermore, an oral challenge with Anisakis death is difficult to apply in clinical practice, and some species-specific secretory molecules may be underrepresented [13].

**Figure 1:**
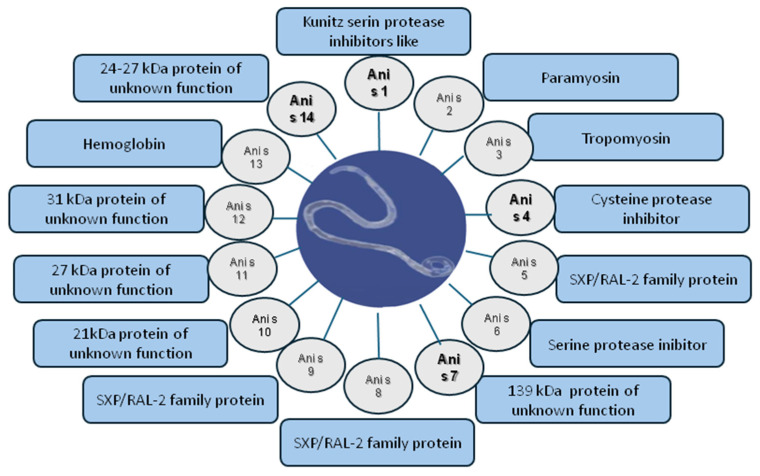
Allergenic molecules of nematode Anisakis with their biological function. The bold text highlights the species-specific allergens.

The allergenic molecules of *Anisakis* can be categorized into three groups and are summarized in Figure 1: (I) molecules indicating true sensitization to the parasite; (II) muscle-origin molecules that cross-react with similar molecules in other species; and (III) molecules belonging to the SXP/RAL protein group, which cross-react with proteins from other parasites [8,14,15,16,17].

Ani s 1, a 24 kDa major species-specific allergen [18,19], is available for diagnostic use on macroarray platforms only, though the recombinant version of the protein does not exhibit the same IgE-binding properties as the natural allergen [20]. IgE binding to Ani s 1 is detected in 60–85% of patients sensitized to *Anisakis*. Ani s 4, a cysteine-protease inhibitor, is another species-specific molecule, recognized by 27–40% of sensitized patients [15,21]. Ani s 7, considered the most important excretory *Anisakis* allergen, is the only allergen recognized in nearly all infected patients and serves as a marker of true contact with the nematode [22,23]. Ani s 14 is a 23.5 kDa protein that induces IgE reactivity in approximately half of the sera from *Anisakis*-allergic patients [24]. Among the somatic allergens, Ani s 2 (paramyosin) has high sequence similarity with paramyosins from other nematodes and arthropods [25]. Ani s 3 (tropomyosin) shares epitopes with homologous proteins from sources such as house dust mites, food, and insects [26,27]. Another major allergen of Anisakis is Ani s 13. It is a hemoglobin and is positive in approximately 60% of patients. However, it is not specific to *Anisakis*, exhibiting cross-reactivity with homologous molecules from other sources [28].

The third group includes Ani s 5, Ani s 8, and Ani s 9, which are minor allergens homologous to nematode proteins in the SXP/RAL-2 protein family. Minor allergens also include Ani s 6, which shows a serine-type endopeptidase inhibitor activity; Ani s 10, a protein of 212 amino acids that has no homology with any other described protein; and Ani s 11 (307 amino acid residues) and Ani s 12 (295 residues). These latter two molecules do not yet have an identified biological function but are recognized by about half of Anisakis-allergic patients [29].

Though no data are yet available on its diagnostic accuracy, ALEX-2 is currently the only system capable of providing information on the primary sensitization source.

Numerous studies have demonstrated that the basophil activation test (BAT) offers high diagnostic accuracy, with specificity close to 100% [30,31,32]. However, it has also yielded positive results in patients with chronic urticaria who benefit from a seafood-free diet [33,34]. We have proposed and validated a diagnostic algorithm to assess whether sensitization to the nematode is present, whether it represents a primary sensitization, and whether it is responsible for the patient’s symptoms [33,34]. The algorithm involves specific IgE testing for Anisakis extracts after ruling out seafood allergies. If positive, further IgE testing for Ascaris and shrimp tropomyosin—key cross-reactive molecules—is conducted. Finally, the clinical relevance of sensitization is evaluated using the BAT.

The aim of this study was to assess the potential role and the accuracy of the multiplex macroarray system in diagnosing *Anisakis* allergy, comparing it with our previously validated diagnostic algorithm [33,34] and evaluating the additional information provided by ALEX-2.

## 2. Results

Thirty-three eligible subjects were recruited for the study from the outpatient clinical centre, including twenty (60.6%) females. Seven (21.2%) of them were in the 0–29 age group, eleven (33.3%) were in the 30–60 age group, and fifteen (45.5%) were over 60 years old. The results of all tested parameters for each patient are summarized in Table 1. Outpatients positive for Ani s 1 plus Ani s 3 comprised 39.39% (95% CI: 22.90–57.86%) of the *Anisakis*-positive subjects. Six out of the nine patients positive for tropomyosin by ImmunoCAP were also positive for tropomyosin Ani s 3 with ALEX-2 (18.18% vs. 27.27% of the thirty-three patients positive on ImmunoCAP; chi-squared = 0.76; *p*-value = 0.38). Among the three samples that were Ani s 3-negative but shrimp tropomyosin-positive by ImmunoCAP, values ranged from 0.69 to 2.09 kIU, and only one was BAT positive.

Regarding the severity of allergic reactions, the Kendall rank correlation coefficient revealed a significant correlation with Ani s 3 positivity (Kendall’s Tau = 0.31 [95% CI: 0.04–0.56]; *p*-value = 0.01) and *Ascaris* positivity (r = 0.35 [95% CI: 0.129 to 0.55], *p*-value = 0.0045). However, there was no significant correlation with Ani s 1 positivity (r = 0.09 [95% CI: −0.28–0.41]; *p*-value = 0.48).

**Figure 2:**
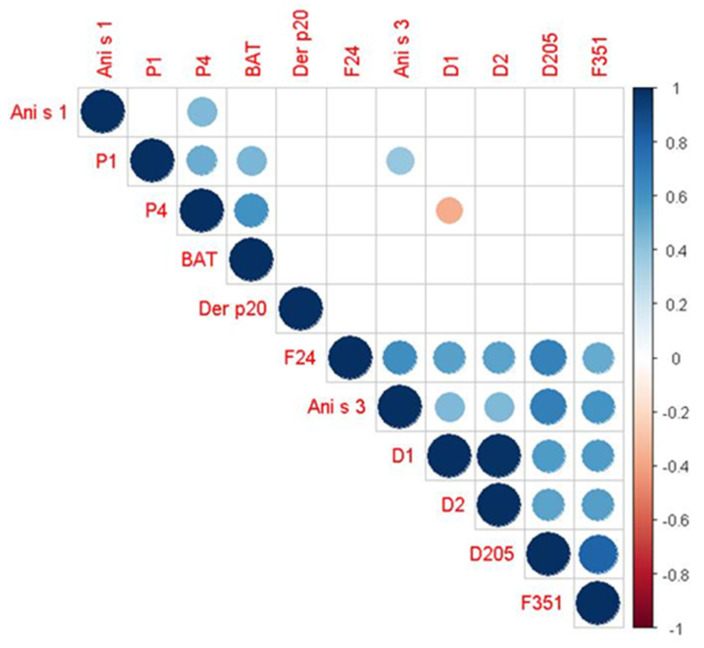
Graphical representation of the correlation matrix using Spearman’s method, showing only statistically significant pairs (*p*-value < 0.05) and arranged by hierarchical clustering (using the “hclust” method). Circles with a blue colour indicate a statistically significant positive (direct) correlation, while those with a red colour indicate a negative (inverse) correlation. The intensity of the colour visually represents the strength of the correlation.

Figure 2 presents a graphical representation of the correlation matrix using Spearman’s approach showing only statistically significant pairs and sorting variables based on hierarchical clustering (“hclust” method). Notable direct correlations were observed between Ani s 1 and p4, p4 and p1, p4 and BAT, p1 and BAT, p1 and Ani s 3, Ani s 3 and f24, f24 and d1, f24 and d2, f24 and d205, f24 and f351, Ani s 3 and d1, Ani s 3 and d2, Ani s 3 and d205, Ani s 3 and f351, d1 and d2, d1 and d205, d1 and f351, d2 and d205, d2 and f351, and d205 and f351. In contrast, an inverse correlation was found between d1 and p4 (*p*-value = 0.038) (Figure 2).

**Figure 3:**
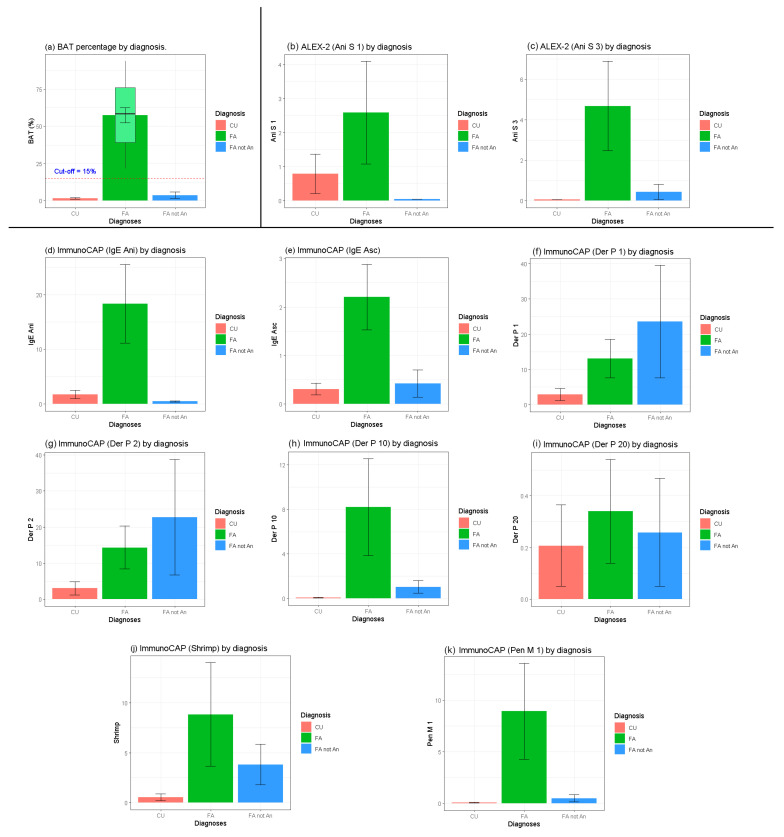
Test results are divided by each diagnostic group. BAT = basophil activation test; Ani s 1 = *Anisakis* major allergen; Ani s 3 = *Anisakis* tropomyosin; Ani = *Anisakis* extract; Asc = *Ascaris* extract; Der p 1 = house dust mite major allergen; Der p 2 = house dust mite major allergen; Der p 10 = house dust mite tropomyosin; Der p 20 = house dust mite arginine kinase; Shrimp = shrimp extract; Pen m 1 = shrimp tropomyosin; FA = food allergy; CU = chronic urticaria.

Logistic regression models were used to explore potential associations with BAT or p4 positivity, adjusting for gender and age. Model A (BAT > 15%, regressor Ani s 1 and Ani s 3) showed no statistically significant association between BAT positivity and Ani s 1 (OR = 1.27 [95% CI: 0.18–9.20]; *p*-value = 1.0) or Ani s 3 positivity (OR = 11.38 [95% CI: 0.79–163.59]; *p*-value = 0.4). In Model B (BAT >15%, regressor p4), a statistically significant association was found between BAT positivity and p4 positivity (OR = 11.77 [95% CI: 1.07–129.16]; *p*-value = 0.04). Finally, in Model C (p4 positivity, regressor Ani s 1 and Ani s 3), neither Ani s 1 positivity (OR = >1000 [95% CI: 0–∞]; *p*-value = 1.0) nor Ani s 3 positivity (OR = 0.72 [95% CI: 0.04–13.07]; *p*-value = 0.8) showed significant associations with *Anisakis* positivity. Figure 3 graphically represents the distribution of values obtained in the tests performed and their statistical significance among the examined patient groups. Specifically, it clearly shows that the levels of activated basophils, specific IgE for *Anisakis* and *Ascaris* extracts, and IgE for tropomyosins (Ani s 3, Der p 10, and Pen m 1) were significantly higher in patients with *Anisakis*-induced food allergy as compared to those with chronic urticaria or non-*Anisakis*-induced food allergy. Moreover, a statistically significant difference in the levels of specific IgE for Ani s 1 was highlighted between patients with non-*Anisakis*-induced food allergy and those with *Anisakis*-induced food allergy, but not in the comparison with patients affected by chronic urticaria.

In Table 2, the statistically significant results are summarized.

A significant correlation is present between BAT and IgE for Ascaris (P1) and Anisakis (P4) and between IgE for tropomyosin (D205, F351, and Ani s 3) and IgE for P4, shrimp (F24), mites (D1-D2), and P1. The correlation is negative between IgE for D1 and P4.

## 3. Discussion

From a molecular perspective, *Anisakis* contains both species-specific epitopes and cross-reactive epitopes shared with somatic molecules from other allergenic sources, such as foods (e.g., shrimp), house dust mites, and other helminths. Therefore, determining whether a positive reaction to the *Anisakis* extract represents primary sensitization or cross-reactivity is crucial. From a diagnostic perspective, the skin prick test can only be performed with an *Anisakis* extract allergen, whose diagnostic performance has not been fully established yet. In vitro IgE assays are available for *Anisakis* extract allergen and another nematode, *Ascaris*. For molecular diagnosis via singleplex methodology, the tropomyosin molecules of *Dermatophagoides pteronyssinus* and shrimp are the only molecules available to date. In contrast, multiplex macroarray platforms like ALEX-2 may allow the detection of specific IgE to Ani s 1 and Ani s 3.

### 3.1. Statistical Evaluation

The correlations observed between the available diagnostic tests provided interesting insights. Notably, the significant correlation between specific IgE for *Anisakis* and *Ascaris*, as well as between specific IgE for *Ascaris* and the BAT, highlighted the potential contribution of other nematodes to clinically relevant *Anisakis* sensitization. This finding was further confirmed by the statistically significant correlation between the severity of the reaction and *Ascaris* and was concordant with previously published evidence regarding the allergic relevance of cross-reactivity between *Ascaris* and *Anisakis* [35,36].

On the other hand, the inverse correlation between IgE levels for *Dermatophagoides pteronyssinus* (house dust mite) and *Anisakis* extracts is intriguing. This may suggest that cross-reactions between mites and Anisakis occur less frequently than previously thought. It is usually considered that cross-reactions occur infrequently with structural identity below 70%, rarely with identity below 50%, and are virtually absent with identity below 30% [37]. It should be emphasized that the correct and precise evaluation of cross-reactions was performed using RAST or ImmunoCAP inhibition tests and specifically for each outpatient. However, this evaluation was not within the scope of this study. Contrary to common assumptions, these data do not support the idea that false positives for *Anisakis* are largely due to cross-reactions with mite antigens.

The correlation between Ani s 1 and *Anisakis* was expected since Ani s 1 is one of the major allergens of the nematode. The major allergens are allergens recognized by specific IgE in more than 50% of individuals allergic to a specific allergenic source. Similarly, the correlation between *Anisakis* and tropomyosins, as well as between tropomyosins from shrimp and mites, was anticipated because these molecules represent significant allergens from these sources. The lack of dependence of Ani s 1 positivity on Ani s 3 that can be inferred as positivity for tropomyosin is more likely to result from sensitization to other allergenic sources mentioned. It is important to emphasize that sensitization to tropomyosin can broaden the range of allergic manifestations to seafood products [38,39]. Consequently, a patient who is clinically allergic to crustaceans might also experience reactions when consuming infested fish. Furthermore, it should be noted that tropomyosin has also been described in vertebrates, particularly in fish such as Tilapia and Atlantic salmon. The molecule Ore m 4, identified as tropomyosin, is an allergen of approximately 33 kDa and was described in a group of patients allergic to Tilapia [39]. It shares 53.5% of structural identity with the homologous molecule in shrimp and, intriguingly, 87.7% of structural identity with the isoform 5 of human tropomyosin. Sixty percent of these patients suffered from inflammatory bowel disease (IBD), suggesting a potential relationship between allergy and IBD. Sal s 4 is a 37 kDa allergenic molecule, also identified as tropomyosin, and is recognized in about one-third of patients allergic to Atlantic salmon [40,41]. These latest observations highlight the need to reassess and improve the diagnostic approach to allergic reactions to seafood products. The link between shrimp and mite allergens was also predictable, as they share many somatic molecules, some of which are major allergens [26,42].

### 3.2. Diagnostic Performance of ALEX-2

Overall, our findings seem to indicate that the ALEX-2, which tests only for Ani s 1 and Ani s 3, is not sufficiently sensitive to be used as a first-line test for diagnosing *Anisakis* allergy. This was predictable, considering that only two of the many nematode molecules are present, and Ani s 7, the most important molecule [22,23,43,44], is missing. A greater number of molecules, or at least the addition of Ani s 7, is necessary to achieve adequate sensitivity. Even better would be the additional inclusion of Ani s 13, as the simultaneous determination of three major allergens would provide significant sensitivity, enabling diagnosis in the majority of patients while maintaining high specificity. When evaluating results from the literature, data on *Anisakis* allergenic molecules are often obtained using ELISA, Western blot, or dot blot methods. Many of these methods are home-made or, when commercially available, are labelled as “for research use only” and not approved for diagnostic use. Consequently, there is a lack of standardization, with significant differences in the diagnostic accuracy among the methods. ELISA, as well as line blot and dot blot methods, may provide high sensitivity but can be less specific, leading to a certain percentage of false positives. Additionally, blots are subject to subjective interpretation. In contrast, more standardized methods, such as ImmunoCAP or the macroarray ALEX-2, are still inadequate for the molecular diagnosis of Anisakis allergy. Thus, serological diagnostics for the parasite still require significant improvement.

When reconsidering the diagnostic methods currently available, however, in cases where Ani s 1 is positive, primary sensitization to *Anisakis* is confirmed. IgE reactivity to Ani s 1 did not correlate with the severity of allergic reactions [45,46,47,48], while a moderate, but statistically significant, correlation was found between Ani s 3 positivity and symptom severity. This finding may align with previous knowledge, as tropomyosin, a thermostable allergen, is known to trigger severe reactions both through inhalation and ingestion [39,42]. However, the above-mentioned moderate correlation did not allow us to exclude that other factors might have influenced the severity of clinical reactions. In addition, it should be clarified that a prognostic indication does not equate to identifying a specific IgE level predictive of a severe reaction. All previous studies have failed to identify a specific IgE cut-off level predictive in this regard [48,49,50].

Interestingly, three patients who tested positive for shrimp and mite tropomyosin were negative for Ani s 3, which could be due to differences in sequence identity between the molecules rather than lower sensitivity of ALEX-2. Nevertheless, it is well documented that the array’s sensitivity is lower compared to ImmunoCAP due to the differences in test architecture [51,52,53]. Additionally, four patients positive for *Anisakis* but negative for Ani s 1 and Ani s 3 were found to be positive for Der p 20 (mite arginine kinase). A homologous molecule with allergenic relevance has been identified in shrimp but has not yet been documented in *Anisakis*. An advantage of the macroarray is that it includes numerous somatic antigens from mites and crustaceans, which may be useful for future studies. In particular, it includes Dermatophagoides pteronyssinus paramyosin (Der p 11). A homologous paramyosin has also been identified in Anisakis, potentially contributing to cross-reactivity. However, data on its clinical relevance remain insufficient. Other potential allergens, such as Myosin Light Chain, are expected to be identified in Anisakis in the future, and the macroarray could be valuable in assessing their prevalence and clinical impact. To distinguish between primary and secondary sensitization, it is useful to compare the quantity of specific IgE detected for crustaceans, mites, and ascarids against that for Anisakis. This ratio, along with clinical history, can help to determine the primary source of sensitization. Currently, known cross-reactions leading to allergic responses to Anisakis—when primary sensitization stems from other sources—primarily involve tropomyosin (mites and crustaceans) and ascarids. In clinical practice, the basophil activation test (BAT) is crucial in these cases, as it closely mimics a challenge test and provides valuable insights into the clinical relevance of laboratory findings.

### 3.3. Limits and Perspectives

Despite these promising preliminary results, the study had several limitations—including its cross-sectional design, the small sample size, and a potential selection bias that must be acknowledged.

Lastly, the diagnosis of *Anisakis* allergy in this study was based on a previously described algorithm [33,34], which includes the BAT to assess clinical relevance. While this method is highly predictive for diagnosing *Anisakis* allergy, it does not predict symptom severity [54], whereas the macroarray seems to offer further insights in this area.

It will also be useful to follow up patients tested with the macroarray to assess its predictive value and long-term effects.

Further studies with larger sample sizes are needed to explore the presence and allergenic significance of arginine kinase in *Anisakis* or other molecules that will be identified in the future.

## 4. Materials and Method

### 4.1. Study Design and Population

An observational cross-sectional study was conducted on a sample of outpatients consecutively enrolled in the Allergy Unit of the “Fatebenefratelli Buccheri La Ferla” Hospital in Palermo (Sicily, Italy), between January and December 2023, with symptoms compatible with *Anisakis* sensitization. Informed consent forms and a previously validated questionnaire [55] were administered to every recruited outpatient to collect socio-demographic and clinical data. Inclusion criteria were a recent history suggestive of IgE sensitization to *Anisakis*, including the presence of at least one of the following symptoms after consuming fresh saltwater fish: asthma, urticaria and/or angioedema, abdominal pain, diarrhea, vomiting, or anaphylaxis. Particular attention has been given to evaluating the cause–effect relationship between symptoms and the consumption of seafood products. Other potential causes capable of producing the same clinical symptoms were excluded. Patients presenting with urticaria for more than six weeks were classified as having chronic urticaria and were included as well, as a portion of these cases might have been related to a potential *Anisakis* sensitization [13,19,22,32]. A documented fish allergy was the exclusion criterion. Those with fish allergy were excluded based on negative specific IgE results for codfish extract and a negative macroarray (ALEX-2) in patients with occasional, non-recurrent reactions to fish. In patients who had not consumed fish post-reaction and tested negative for specific IgE, a prick-by-prick test was conducted with the suspected fish.

The study was conducted in accordance with the Declaration of Helsinki and was approved by the Palermo Ethics Committee 1, Italy (protocol n. 8/2018).

### 4.2. Laboratory Analyses

Following a previously validated comprehensive diagnostic algorithm [33,34], first-line testing included the skin prick test (SPT) for *Anisakis* and codfish extracts. Specific IgE levels for codfish (f3) and *Anisakis* (p4) were measured using the ImmunoCAP 250 system (Thermo Fisher Diagnostics, Uppsala, Sweden), with a cut-off value of >0.10 kIU/L considered positive [35]. For second-line testing, patients who were negative for fish tests but positive for *Anisakis* with SPT or ImmunoCAP underwent specific IgE testing for *Dermatophagoides pteronyssinus* (d1), *Dermatophagoides farinae* (d2), shrimp extracts (f24), *Ascaris* (p1), tropomyosins from shrimp (f351), and *Dermatophagoides pteronyssinus* (d205).

All sera were tested using the macroarray ALEX-2 system (Macro Array Diagnostics, Vienna, Austria). Patients who tested positive for *Anisakis* were further evaluated using the basophil activation test (BAT) as a clinical confirmatory analysis. BAT was performed using the Flow CAST kit (Bühlmann Laboratories AG, Schönenbuch, Switzerland) and home-made *Anisakis* extracts obtained from *Anisakis pegreffii*, used at a concentration of 22.5 ng/mL, as proposed in previous studies [33,34]. Cut-off values of >15% of activated basophils, and a stimulation index (activated/resting basophils) > 2, were used [33].

### 4.3. Statistical Analysis

The following variables were considered in the statistical analyses: sex, age at sampling, specific IgE to p4, p1, d1, d2, d205, f24, f351, BAT, Ani s 1, and Ani s 3, diagnosis (*Anisakis* food allergy, chronic spontaneous urticaria, non-*Anisakis* food allergy), and severity of allergic reaction (“0” = none; “1” = non-anaphylaxis; “2” = anaphylaxis).

Spearman’s correlation test was used to assess bivariate correlations among the variables, with a graphical representation of significant associations in a correlation matrix. Discrepancies between outcomes based on different cut-offs for the same variable were evaluated using the McNemar test, while the agreement between positivity for various reagents was assessed using Cohen’s k correlation index. Cross-positivity between f351, d205, and Ani s 3 was analyzed through percentage distribution across the sample.

Logistic regression models were applied to assess the association of Ani s 1 and Ani s 3 (in various combinations) as well as p4 with BAT positivity. Kendall’s rank correlation coefficient was used to evaluate the relationship between Ani s 1, Ani s 3, *Ascaris*, and the severity of allergic reactions.

## 5. Conclusions

In conclusion, our preliminary results suggested that ALEX-2 might not be sufficiently sensitive to be used as a first-line test for *Anisakis* allergy diagnosis. Nevertheless, it could provide valuable additional information, since positivity for Ani s 1 indicates a primary sensitization to the nematode, while Ani s 3 positivity seems to correlate with the severity of the clinical reactions, which BAT cannot predict.

More structured prospective longitudinal studies with larger samples are needed to confirm these findings and to better evaluate the predictive value of ALEX-2 in diagnosing Anisakis allergy.

## Figures and Tables

**Table 1:** Results of the allergological test of each individual outpatient. SPT = skin prick test for Anisakis extracts; BAT Anisakis = % of activated basophils; FA = food allergy; FA = a food allergy not triggered by Anisakis; CU = chronic urticaria.

				Specific IgE kIU/L									ALEX 2 kIU/L	
Patients	SPT	Anisakis	Ascaris	Dermat. P	Dermat. F	Tropom.Mites	Shrimp	Tropom.Shrimp	BAT Anisakis	Diagnosis	Grade	Ani s 1	Ani s 3	Der p 20
1	+	1.91	0.15	3.56	3.73	0.08	0	0.83	24.44	FA	1	<0.10	<0.10	<0.10
2	−	0.42	0.84	0.07	0.05	0.01	0	0	3.15	CU	0	<0.10	<0.10	<0.10
3	+	2.29	0.18	1.05	1.39	0.03	0.84	0	2.36	CU	0	<0.10	<0.10	<0.10
4	−	0.19	0.04	97.8	100	0.07	0.04	0.03	1.46	FA not An	1	<0.10	<0.10	<0.10
5	+	100	7.27	0.03	0.05	0.05	0.09	0.03	93.72	FA	1	5.35	<0.10	<0.10
6	+	2.31	3.01	6.21	5.47	9.87	12.5	10.3	36.58	FA	1	<0.10	9.76	3.26
7	+	5.4	0.47	0.86	0.47	0.1	0.26	0.1	0.69	CU	0	1.19	<0.10	<0.10
8	+	98.3	6	0.03	0.02	0.1	0.09	0.1	84.43	FA	2	<0.10	<0.10	<0.10
9	−	1.04	1.8	1.66	2.4	0.1	7.52	0.1	0.21	FA not An	1	<0.10	<0.10	<0.10
10	+	3.99	6.16	41.5	43.4	31.4	27.9	30.27	74.07	FA	2	<0.10	21.07	<0.10
11	+	1.92	0.15	0.53	0.57	0.01	0.79	0.01	21.57	FA	1	<0.10	<0.10	2.63
12	−	0.21	0.55	6.78	6.69	0.11	2.53	0.14	0.2	CU	0	<0.10	<0.10	<0.10
13	−	0.06	0.06	0.36	0.07	0.01	0.6	0.01	39.52	FA	1	<0.10	<0.10	<0.10
14	−	0.67	3.44	12.4	10.25	0.03	0.83	0.05	30.63	FA	1	<0.10	<0.10	<0.10
15	−	0.53	0.16	37.3	27.5	0.75	0.32	0.69	0.97	FA not An	1	<0.10	<0.10	<0.10
16	+	7.3	0.58	16.9	21	39.2	29.2	51	41.24	FA	1	0.26	27.92	<0.10
17	−	0.43	2.24	0.07	0.09	0.01	0.01	0.03	48	FA	1	<0.10	<0.10	<0.10
18	−	0.32	0.05	0.9	0.77	0.01	0.91	0.03	13.4	FA not An	1	<0.10	<0.10	1.3
19	−	0.49	0.37	0.88	0.91	1.89	12.3	2.09	5.19	FA not An	1	<0.10	<0.10	<0.10
20	+	3.2	0.07	0.04	0.09	0.01	0.03	0.05	3.15	CU	0	4.09	<0.10	<0.10
21	−	0.24	0.09	3.11	4.71	3.42	1.81	<0.10	1.01	FA not An	1	<0.10	2.34	<0.10
22	−	0.36	0.02	5.53	4.02	0.05	0.05	4.18	42.4	FA	1	4.09	<0.10	<0.10
23	+	19.1	0.72	0.02	0.03	0	0	0.04	74.8	FA	1	<0.10	<0.10	<0.10
24	−	0.38	0.05	0.04	0.03	0.05	0.05	0.02	0.35	CU	0	<0.10	<0.10	<0.10
25	+	4.09	10.5	100	100	76.6	100	78.3	81.3	FA	2	4.56	31.14	<0.10
26	−	0.35	0	11.6	12.5	0.28	0.14	0.23	1.12	CU	0	<0.10	<0.10	1.15
27	+	15	1.89	0.01	0.01	0.01	0.06	0.01	74.6	FA	2	<0.10	<0.10	<0.10
28	+	74.2	0.29	0.06	0.03	0.06	0.05	0.01	58.9	FA	2	29.67	<0.10	<0.10
29	+	2.35	0.08	35.58	32.14	0.01	2.58	0.01	61.27	FA	1	<0.10	<0.10	<0.10
30	+	12.4	0.11	0.01	0.01	0	0	0.01	58	FA	1	<0.10	<0.10	<0.10
31	−	0.36	1.01	3.24	2.97	6.25	1.92	3.17	79.67	FA	1	<0.10	2.93	<0.10
32	+	14	0.2	0.14	0.16	0	0.01	0	85.3	FA	1	7.19	<0.10	<0.10
33	+	7.35	0.19	35.9	62.2	0.01	0.05	0.05	37.67	FA	1	<0.10	<0.10	<0.10

**Table 2:** Correlation matrix using Spearman’s method, showing statistically significant pairwise correlations only (*p*-value < 0.05).

	Spearman’s *ρ* (*p*-Value)							
Variables	P1	P4	Ani S3	D1	D2	D205	F24	F351
P1	–	+49.7% (0.003)	–	–	–	–	–	–
BAT	+45.0% (0.009)	+60.7% (<0.001)	–	–	–	–	–	–
Ani S1	–	+44.3% (0.010)	–	–	–	–	–	–
Ani S3	+38.9% (0.025)	–	–	+44.3% (0.010)	+44.3% (0.010)	+68.0% (<0.001)	+61.6% (<0.001)	+59.5% (<0.001)
D1	–	−36.1% (0.038)	+44.3% (0.010)	–	+98.6% (<0.001)	+56.8% (<0.001)	54.8% (<0.001)	+56.1% (<0.001)
D2	–	–	+44.3% (0.010)	+98.6% (<0.001)	–	+53.8% (0.001)	+53.6% (0.001)	+55.7% (<0.001)
D205	–	–	+68.0% (<0.001)	+56.8% (<0.001)	+53.8% (0.001)	–	+67.7% (<0.001)	+80.5% (<0.001)
F24	–	–	+61.6% (<0.001)	54.8% (<0.001)	+53.6% (0.001)	+67.7% (<0.001)	–	+50.6% (0.003)
F351	–	–	+59.5% (<0.001)	+56.1% (<0.001)	+55.7% (<0.001)	+80.5% (<0.001)	+50.6% (0.003)	–

P1 = *Ascaris*; P4 = *Anisakis*; BAT = basophil activation test; D1 = *Dermatophagoides Pteroronisssinus*; D2 = *Dermatophagoides Farinae*; D205 = house dust mite tropomyosin; F24 = shrimp; F351= shrimp tropomyosin

## Data Availability

The data presented in this study are available upon request from the corresponding author. The data are not publicly available due to privacy reasons.

## References

[1] Smith J.W., Wootten R. (1987). Anisakis and anisakiasis. Adv. Parasitol..

[2] World Health Organization Soil-Transmitted Helminths. https://www.who.int/news-room/fact-sheets/detail/soil-transmitted-helminth-infections.

[3] Pravettoni V., Primavesi L., Piantanida M. (2012). *Anisakis simplex*: Current knowledge. Eur. Ann. Allergy Clin. Immunol..

[4] Baird F.J., Gasser R.B., Jabbar A., Lopata A.L. (2014). Foodborne anisakiasis and allergy. Mol. Cell Probes.

[5] Tejada M., Olivares F., de las Heras C., Careche M., Solas M.T., García M.L., Fernandez A., Mendizábal A., Navas A., Rodríguez-Mahillo A.I. (2015). Antigenicity of *Anisakis simplex* s.s. L3 in parasitized fish after heating conditions used in the canning processing. J. Sci. Food Agric..

[6] Mazzucco W., Raia D.D., Marotta C., Costa A., Ferrantelli V., Vitale F., Casuccio A. (2018). Anisakis sensitization in different population groups and public health impact: A systematic review. PLoS ONE.

[7] Mazzucco W., Lacca G., Cusimano R., Provenzani A., Costa A., Di Noto A.M., Massenti M.F., Leto-Barone M.S., Lorenzo G.D., Vitale F. (2012). Prevalence of sensitisation to *Anisakis simplex* among professionally exposed populations. Arch. Environ. Occup. Health.

[8] Carballeda-Sangiao N., Olivares F., Rodriguez-Mahillo A.I., Careche M., Tejada M., Moneo I., González-Muñuoz M. (2014). Identification of autoclave-resistant *Anisakis simplex* allergens. J. Food Prot..

[9] Petithory J.C. (2007). New data on anisakiasis. Bull. Acad. Natl. Med..

[10] Pozo M.D., Audicana M., Diez J.M., Munoz D., Ansotegui I.J., Fernández E., García M., Etxenagusia M., Moneo I., de Corres L.F. (1997). *Anisakis simplex*, a relevant etiologic factor in acute urticaria. Allergy.

[11] Daschner A., De Frutos C., Valls A., Vega F. (2010). *Anisakis simplex* sensitization-associated urticaria: Short-lived immediate type or prolonged acute urticaria. Arch. Dermatol. Res..

[12] Sastre J., Lluch-Bernal M., Quirce S., Arrieta I., Lahoz C., Del Amo A., Fernández-Caldas E., Marañón F. (2000). A double blind, placebo-controlled oral challenge study with lyophilized larvae and antigen of the fish parasite, *Anisakis simplex*. Allergy.

[13] Ventura M.T., Napolitano S., Menga R., Cecere R., Asero R. (2013). *Anisakis simplex* hypersensitivity is associated with chronic urticaria in endemic areas. Int. Arch. Allergy Immunol..

[14] Kobayashi Y., Ikeda K., Shiomi K. (2010). Elucidation of IgE-binding epitopes of Ani s 1: The major Anisakis simplex allergen. J. Mol. Biochem. Parasitol..

[15] Caballero M.L., Asero R., Antonicelli L., Kamberi E., Colangelo C., Fazii P., De Burgos C., Rodriguez-Perez R. (2013). Anisakis allergy component-resolved diagnosis: Clinical and immunologic differences between patients from Italy and Spain. Int. Arch. Allergy Immunol..

[16] Lin A.H., Nepstad I., Florvaag E., Egaas E., van Do T. (2014). An extended study of seroprevalence of anti-Anisakis simplex IgE antibodies in Norwegian blood donors. J. Scand. J. Immunol..

[17] Cuéllar C., Daschner A., Valls A., De Frutos C., Fernández-Fígares V., Anadón A.M., Rodríguez E., Gárate T., Rodero M., Ubeira F.M. (2012). Ani s 1 and Ani s 7 recombinant allergens are able to differentiate distinct *Anisakis simplex*-associated allergic clinical disorders. Arch. Dermatol. Res..

[18] Moneo I., Caballero M.L., Gómez F., Ortega E., Alonso M.J. (2000). Isolation and characterization of a major allergen from the fish parasite *Anisakis simplex*. J. Allergy Clin. Immunol..

[19] Toro C., Caballero M.L., Baquero M., Garcia-Samaniego J., Casado I., Rubio M., Moneo I. (2004). High Prevalence of Seropositivity to a Major Allergen of *Anisakis simplex*, Ani s 1, in Dyspeptic Patients. Clin. Diagn. Lab. Immunol..

[20] Shimakura K., Miura H., Ikeda K., Ishizaki S., Nagashima Y., Shirai T., Kasuya S., Shiomi K. (2004). Purification and molecular cloning of a major allergen from *Anisakis simplex*. Mol. Biochem. Parasitol..

[21] Moneo I., Caballero M.L., Gonzalez-Munoz M., Rodriguez-Mahillo A.I., Rodriguez-Perez R., Silva A. (2005). Isolation of a heat-resistant allergen from the fish parasite *Anisakis simplex*. Parasitol. Res..

[22] Rodríguez E., Anadón A.M., García-Bodas E., Romarís F., Iglesias R., Gárate T., Ubeira F.M. (2008). Novel sequences and epitopes of diagnostic value derived from the *Anisakis simplex* Ani s 7 major allergen. Allergy.

[23] Anadon A.M., Romaris F., Escalante M., Rodriguez E., Garate T., Cuellar C., Ubeira F.M. (2009). The *Anisakis simplex* Ani s 7 major allergen as an indicator of true *Anisakis* infections. Clin. Exp. Immunol..

[24] Kobayashi Y., Kakemoto S., Shimakura K., Shiomi K. (2015). Molecular cloning and expression of a new major allergen, Ani s 14, from *Anisakis simplex*. Shokuhin Eiseigaku Zasshi.

[25] Perez-Perez J., Fernandez-Caldas E., Maranon F., Sastre J., Bernal M.L., Rodriguez J. (2000). Molecular cloning of paramyosin, a new allergen of *Anisakis simplex*. Int. Arch. Allergy Immunol..

[26] Kochanowski M., Różycki M., Dąbrowska J., Bełcik A., Karamon J., Sroka J., Cencek T. (2020). Proteomic and Bioinformatic Investigations of Heat-Treated *Anisakis simplex* Third-Stage Larvae. Biomolecules.

[27] Fæste C.K., Jonscher K.R., Dooper M.M., Egge-Jacobsen W., Moen A., Daschner A., Egaas E., Christians U. (2014). Characterisation of potential novel allergens in the fish parasite *Anisakis simplex*. EuPA Open Proteom..

[28] González-Fernández J., Daschner A., Nieuwenhuizen N.E., Lopata A.L., De Frutos C., Valls A., Cuéllar C. (2015). Haemoglobin, a new major allergen of *Anisakis simplex*. Int. J. Parasitol..

[29] Fitzsimmons C.M., Falcone F.H., Dunne D.W. (2014). Helminth allergens, parasite-specific IgE, and its protective role in human immunity. Front. Immunol..

[30] Gamboa P.M., Asturias J., Martínez R., Antépara I., Jáuregui I., Urrutia I., Fernández J., Sanz M.L. (2012). Diagnostic utility of components in allergy to *Anisakis simplex*. J. Investig. Allergol. Clin. Immunol..

[31] Gonzalez-Muñoz M., Luque R., Nauwelaers F., Moneo I. (2005). Detection of *Anisakis simplex*-induced basophil activation by flow cytometry. Cytometry B Clin. Cytom..

[32] Frezzolini A., Cadoni S., De Pita O. (2010). Usefulness of the CD63 basophil activation test in detecting *Anisakis* hypersensitivity in patients with chronic urticaria: Diagnosis and follow-up. Clin. Exp. Dermatol..

[33] Brusca I., Graci S., Barrale M., Cammilleri G., Zarcone M., Onida R., Costa A., Ferrantelli V., Buscemi M.D., Uasuf C.G. (2020). Use of a comprehensive diagnostic algorithm for *Anisakis* allergy in a high seroprevalence Mediterranean setting. Eur. Ann. Allergy Clin. Immunol..

[34] Brusca I., Barrale M., Zarcone M., Fruscione S., Onida R., De Bella D.D., Alba D., Belluzzo M., Uasuf C.G., Cammilleri G. (2023). Basophil Activation Test in the Diagnosis of *Anisakis* Allergy: An Observational Study from an Area of High Seafood Consumption in Italy. Pathogens.

[35] Lanfranchi A.L., Sardella N.H. (2010). *Anisakis* survival after microwaving, freezing and salting fish from Argentina. Food Sci. Technol. Res..

[36] Audicana M.T., Ansotegui I.J., de Corres L.F., Kennedy M.W. (2002). *Anisakis simplex*: Dangerous—Dead and alive?. Trends Parasitol..

[37] FAO/WHO (2002). Evaluation of Allergenicity of Genetically Modified Foods. Report of a Joint FAO/WHO Expert Consultation on Allergenicity of Foods from Biotechnology.

[38] Ruethers T., Taki A.C., Johnston E.B., Nugraha R., Le T.T., Kalic T., McLean T.R., Kamath S.D., Lopata A.L. (2018). Seafood allergy: A comprehensive review of fish and shellfish allergens. Mol. Immunol..

[39] Peixoto S., Monteiro T., Carvalho M., Santos M., Matos C., Bartolomé B., Labrador-Horrillo M., Quaresma M. (2018). Vertebrate Tropomyosin as an Allergen. J. Investig. Allergol. Clin. Immunol..

[40] Liu R., Holck A.L., Yang E., Liu C., Xue W. (2013). Tropomyosin from tilapia (*Oreochromis mossambicus*) as an allergen. Clin. Exp. Allergy.

[41] Ruethers T., Taki A.C., Karnaneedi S., Nie S., Kalic T., Dai D., Daduang S., Leeming M., Williamson N.A., Breiteneder H. (2021). Expanding the allergen repertoire of salmon and catfish. Allergy.

[42] Celi G., Brusca I., Scala E., Villalta D., Pastorello E., Farioli L., Cortellini G., Deleonardi G., Galati P., Losappio L. (2020). House dust mite allergy and shrimp allergy: A complex interaction. Eur. Ann. Allergy Clin. Immunol..

[43] de Las Vecillas L., Muñoz-Cacho P., López-Hoyos M., Monttecchiani V., Martínez-Sernández V., Ubeira F.M., Rodríguez-Fernández F. (2020). Analysis of Ani s 7 and Ani s 1 allergens as biomarkers of sensitization and allergy severity in human anisakiasis. Sci. Rep..

[44] Cardona V., Ansotegui I.J. (2016). Component-resolved diagnosis in anaphylaxis. Curr. Opin. Allergy Clin. Immunol..

[45] Aibinu I.E., Smooker P.M., Lopata A.L. (2019). *Anisakis* nematodes in fish and shellfish—From infection to allergies. Int. J. Parasitol. Parasites Wildl..

[46] Armentia A., Santos J., Serrano Z., Martín B., Martín S., Barrio J., Fernández S., González-Sagrado M., Pineda F., Palacios R. (2017). Molecular diagnosis of allergy to *Anisakis simplex* and *Gymnorhynchus gigas* fish parasites. Allergol. Immunopathol..

[47] González-Fernández J., Rivas L., Luque-Ortega J.R., Núñez-Ramírez R., Campioli P., Gárate T., Perteguer M.J., Daschner A., Cuéllar C. (2017). Recombinant vs. native *Anisakis* haemoglobin (Ani s 13): Its appraisal as a new gold standard for the diagnosis of allergy. Exp. Parasitol..

[48] Yang A.C., Arruda L.K., Santos A.B.R., Barbosa M.C., Chapman M.D., Galvão C.E., Kalil J., Morato-Castro F.F. (2010). Measurement of IgE antibodies to shrimp tropomyosin is superior to skin prick testing with commercial extract and measurement of IgE to shrimp for predicting clinically relevant allergic reactions after shrimp ingestion. J. Allergy Clin. Immunol..

[49] Sampson H.A. (2001). Utility of food-specific IgE concentrations in predicting symptomatic food allergy. J. Allergy Clin. Immunol..

[50] Sampson H.A., Aceves S., Bock S.A., James J., Jones S., Lang D., Nadeau K., Nowak-Wegrzyn A., Oppenheimer J., Perry T.T. (2014). Food allergy: A practice parameter update—2014. J. Allergy Clin. Immunol..

[51] Ukleja-Sokołowska N., Lis K., Żbikowska-Gotz M., Adamczak R., Kuźmiński A., Bartuzi Z. (2021). Clinical utility of immunological methods based on the singleplex and multiplex ImmunoCap systems for diagnosis of shrimp allergy. J. Int. Med. Res..

[52] Foong R.X., Roberts G., Fox A.T., du Toit G. (2016). Pilot study: Assessing the clinical diagnosis of allergy in atopic children using a macroarray assay in addition to skin prick testing and serum specific IgE. Clin. Mol. Allergy.

[53] Buzzulini F., Da Re M., Scala E., Martelli P., Conte M., Brusca I., Villalta D. (2019). Evaluation of a new multiplex assay for allergy diagnosis. Clin. Chim. Acta.

[54] Cañas J.A., Pérez-Sánchez N., Lopera-Doblas L., Palomares F., Molina A., Bartra J., Torres M.J., Gómez F., Mayorga C. (2022). Basophil Activation Test Utility as a Diagnostic Tool in LTP Allergy. Int. J. Mol. Sci..

[55] Fruscione S., Barrale M., Zarcone M., Alba D., Ravazzolo B., Belluzzo M., Onida R., Cammilleri G., Costa A., Ferrantelli V. (2024). Screening of *Anisakis*-Related Allergies and Associated Factors in a Mediterranean Community Characterized by High Seafood Consumption. Foods.

